# Changes of Intestinal Flora in Patients with Atrial Fibrillation and Its Correlation with Cardiovascular Risk Factors

**DOI:** 10.31083/j.rcm2404110

**Published:** 2023-04-17

**Authors:** Shi Chen, Mingyue Tu, Jiaran Shi, Xiaosheng Hu

**Affiliations:** ^1^Department of General Practice, Shulan (Hangzhou) Hospital Affiliated to Zhejiang Shuren University, Shulan International Medical College, 310000 Hangzhou, Zhejiang, China; ^2^Department of Cardiology, The First Affiliated Hospital, College of Medicine, Zhejiang University, 310000 Hangzhou, Zhejiang, China

**Keywords:** atrial fibrillation, intestinal flora, 16S rDNA, CHA_2_DS_2_-VAS_C_ score

## Abstract

**Background:**

Based on the 16S rDNA sequence, intestinal flora changes in atrial fibrillation (AF) patients were monitored, the correlation between the changes and ${\mathrm{CHA}}_{2}$${\mathrm{DS}}_{2}$-${\mathrm{VAS}}_{\text{C}}$ score was analyzed, and the possible related factors affecting the changes of intestinal flora were investigated.

**Methods:**

According to the inclusion criteria, 53 AF patients were selected as atrial fibrillation group (Group AF), detection of C-reactive protein (CRP), homocysteine (Hcy), total bile acid (TBA), brain natriuretic peptide (BNP), High-sensitivity cardiac troponin (Hs-cTn) and left ventricular ejection fraction (LVEF) were accomplished. A total of 29 healthy subjects who underwent physical examination with matched gender and age were selected as the healthy group (Group H), and the same examinations as in Group AF were handled. Structural composition of intestinal flora was detected and analyzed by 16S rRNA sequencing technology. Flora differences between Group AF and Group H were counted, and the correlation analysis among age, Hs-cTn, CRP, TBA, Hcy, BNP and LVEF were explored. Meanwhile, ${\mathrm{CHA}}_{2}$${\mathrm{DS}}_{2}$-${\mathrm{VAS}}_{\text{C}}$ score of 53 AF patients was fulfilled, then patients were divided into three subgroups according to different scores, namely: 0 point (AF-0, n = 9), 1 point (AF-1, n = 15), ≥2 points (AF-2, n = 29). Finally, the correlation of intestinal flora differences and ${\mathrm{CHA}}_{2}$${\mathrm{DS}}_{2}$-${\mathrm{VAS}}_{\text{C}}$ scores were analyzed.

**Results:**

In terms of Alpha diversity, compared with the control group, the abundance and diversity of flora in Group AF were observably reduced. However, at phylum and class level, there was no notable difference in community structure between Group AF and Group H (*p >* 0.05). Further statistics revealed that the composition and abundance of intestinal flora in Group AF were prominently different from those in Group H at phylum, class, order and family levels, which were correlated with CRP and LVEF. Additionally, bioinformatics analysis comparison was performed on three ${\mathrm{CHA}}_{2}$${\mathrm{DS}}_{2}$-${\mathrm{VAS}}_{\text{C}}$ score subgroups of Group AF with Group H. It was reported that at phylum level, the relative abundance of Firmicutes in Group AF-2 and Chloroflexi in Group H was higher. At class level, the relative abundance of Sphingobacteriia, Flavobacteriia and Alphaproteobacteria was higher in group H. At order level, the relative abundance of Sphingobacteriales, Micrococcales, Flavobacteriales, Sphingobacteriales and Rhizobiales in group H was higher. At family level, the relative abundance of Sphingobacteriaceae, Flavobacteriaceae and Clostridiaceae in group H was higher. At genus level, the relative abundance of *Sphingobacterium* in group H, *Clostridiumsensustricto-1* in Group AF-2, *Dialister* and *Allisonella* in Group AF-1, and *Prevotella-9* in Group AF-0 were higher.

**Conclusions:**

There were changes in the relative abundance of intestinal flora at phylum, class, order and family levels, which was concerned with LVEF and CRP value, whereas Alpha diversity index of the flora decreased. The composition and relative abundance of intestinal flora varied in AF patients with ${\mathrm{CHA}}_{2}$${\mathrm{DS}}_{2}$-${\mathrm{VAS}}_{\text{C}}$ scores of 0, 1, and ≥2.

## 1. Introduction

Atrial fibrillation (AF) is the most prevalent arrhythmia, and the prevalence is about 6.5% in people aged ≥65 years [1]. It not only increases the risk of stroke, heart failure and death [2], but also greatly saddle patients with economic burden. It is estimated that the disease burden of AF in China is as high as Chinese Yuan (CNY) 4.9 billion [3]. There are many theories about the mechanism of AF, among which inflammatory response is a considerable one. Various degrees of inflammatory response have been found in the myocardial biopsy of AF patients, such as inflammatory infiltration, myocyte necrosis and fibrosis; many inflammatory factors and mediators, such as C-reactive protein (CRP), interleukin-2 (IL-2), interleukin-6 (IL-6), interleukin-8 (IL-8) and tumor necrosis factor-α (TNF-α), are associated with the occurrence and maintenance of AF.

There are more than 1000 species bacteria residing in human gastrointestinal tract, reaching a number of 10 [4]. Among which intestinal flora is a key regulator of human metabolism, affecting material absorption and energy metabolism, and playing a crucial part in inflammation and immunity [5]. Intestinal flora metabolizes host food into a series of metabolites, including Trimethylamine oxide (TMAO), short-chain fatty acids, branched-chain amino acids, lipopolysaccharide, etc. Many studies have investigated the role of these metabolites in the development and progression of cardiovascular diseases [6]. A key product of gut microbiota is trimethylamine (TMA), which is produced by dietary choline and carnitine (from meat and dairy products) and oxidized to TMAO by fetal hepatic flavin-containing monooxygenase (FMO). Positively correlated with the occurrence of many cardiovascular diseases, such as hypertension, atherosclerosis, coronary heart disease and metabolic syndrome [7], it is a new biomarker for heart disease. Studies of AF and intestinal flora are still in their infancy. Zhang *et al*. [8] indicated that intestinal flora metabolite TMAO is closely related to the occurrence of AF in patients with coronary heart disease, but the mechanism is unclear. In recent years, Yu *et al*. [9] believed that intestinal flora and AF are linked via the intestinal flora-TMAO-inflammatory factor-AF pathway. In the preliminary study, Chen *et al*. [10] found that there was intestinal flora imbalance in elderly AF patients, while inflammatory factors such as CRP and homocysteine (Hcy) were correlated with the number of flora in them. This study, based on 16S rDNA sequence detection, bioinformatics analysis was performed at phylum, class, order, family and genus levels, including operational taxonomic unit (OTU) clustering and species annotation, Alpha diversity, species composition analysis, comparative analysis (Beta diversity) and differential analysis, and the characteristics of intestinal microflora in AF patients were observed. The flora differences between patients with AF and healthy controls were statistically analyzed. The correlation analyses of flora differences and age, inflammatory factors (CRP and Hcy), as well as cardiovascular markers (High-sensitivity cardiac troponin (Hs-cTn), total bile acid (TBA), brain natriuretic peptide (BNP) and left ventricular ejection fraction (LVEF)) were performed, so as to provide the basis for the role of intestinal flora in the inflammatory response mechanism of AF.

${\mathrm{CHA}}_{2}$${\mathrm{DS}}_{2}$-${\mathrm{VAS}}_{\text{C}}$ score is utilized to predict the risk of stroke in patients with non-valvular AF, which is also an independent risk factor for recurrence of AF after returning to sinus rhythm [11]. Our previous study also detected [12] that intestinal flora in elderly patients with high-risk non-valvular AF was closely related to ${\mathrm{CHA}}_{2}$${\mathrm{DS}}_{2}$-${\mathrm{VAS}}_{\text{C}}$. Therefore, we analyzed the characteristics of fecal flora between different ${\mathrm{CHA}}_{2}$${\mathrm{DS}}_{2}$-${\mathrm{VAS}}_{\text{C}}$ scores in AF patients by 16S rRNA and explored possible correlated factors affecting the changes in the microflora.

## 2. Objects and Methods

### 2.1 Research Cohort

53 AF patients who were admitted to the Department of Cardiology of The First Affiliated Hospital, College of Medicine, Zhejiang University from January 2021 to May 2021 and met the inclusion criteria were selected as the Group AF. In Group AF, there were 32 males and 21 females, with an average age of 63.57 (40–74) years old, including 15 patients who drank alcohol, 17 patients who smoked, and 6 patients with stroke, 9 patients with coronary heart disease or arterial vascular stenosis/compound aortic plaque/peripheral arterial disease, 6 cases with diabetes, and 11 cases with recent heart failure, 34 patients with hypertension, 29 with paroxysmal AF, 24 patients with persistent AF, 2 with elevated Hs-cTn, 2 patients with elevated CRP, 8 with elevated TBA, and 1 with elevated Hcy, 25 with elevated BNP, 4 with decreased LVEF, and 42 cases with cardiac dilatation. Furthermore, in line with the ${\mathrm{CHA}}_{2}$${\mathrm{DS}}_{2}$-${\mathrm{VAS}}_{\text{C}}$ score [13], Group AF was divided into three subgroups: 0 point (AF-0, n = 9), 1 point (AF-1, n = 15) and ≥2 points (AF-2, n = 29), and the correlations between each subgroup and intestinal flora changes were analyzed. In the healthy group (Group H), 29 healthy subjects who underwent physical examination were selected, including 18 males and 11 females, with an average age of 62.77 (45–74) years. There were no statistically significant differences in gender and age between Group AF and Group H (*p >* 0.05).

Inclusion criteria: (1) Patients with a history of AF who have been diagnosed by 12-lead electrocardiogram (ECG) or 24-hour dynamic electrocardiogram (DCG). (2) With the approval of Hospital Ethics Committee and with the consent of patients and their families, informed consent was agreed and signed by patients or legal representatives. Exclusion criteria: (1) Age >80 years old, <18 years old. (2) Patients who were unable or unwilling to adhere to standard treatment or did not agree to participate in the test. (3) Diarrhea or other gastrointestinal diseases within the last one month. (4) Those who took probiotics, antibiotics or hormone drugs within the last one month.

### 2.2 Fecal Sample Collection and DNA Extraction

Fresh fecal samples were collected from each patient and immediately frozen at –20 ℃. Samples were then transported on ice to the laboratory, where they were stored at –80 ℃. Fast DNA SPIN Kit for Feces (116540600, MP Biomedicals, Santa Ana, CA, USA) was used to isolate bacterial DNA.

### 2.3 16S rRNA Gene Amplification and Sequencing

V3–V4 region of bacterial 16S ribosomal RNA gene was amplified by polymerase chain reaction (PCR). Primer sequences were as follows: 341F 5′-Barcode-CCTAYGGGRBGCASCAG-3′ and 806R 5′-GGACTACNNGGGTATCTAAT-3′. Purification and quantification were then performed using the Axyprep DNA gel extraction kit (CB59718017, Axygen Biosciences, Union City, CA, USA) and Quantifluor-ST (QUANTIFLUOR™ST/P, Promega, Madison, WI, USA). Finally, the purified amplicons were sequenced (2 × 250 bp) on the Illumina novaseq 6000 sequencing instrument (MKBio Co., Ltd, Hangzhou, China). OTU clustering of sequencing data was performed in Usearch (vsesion 10, http://drive5.com/uparse/) in accordance with 97% similarity. Then, species annotations are made with Uclust and Silva (version 132, Max Planck Institute for Marine Microbiology and Jacobs University, Bremen, Germany) database, at a 70% confidence threshold, to obtain the abundance table of OTU and taxonomic levels, as well as the corresponding phylogenetic tree.

### 2.4 Blood Sample Collection and Detection

The fasting peripheral blood of patients in Group AF was collected in the morning and sent to the laboratory, and plasma CRP, TBA and Hcy levels were measured by Hitachi 7600 automatic biochemical analyzer. Getein1600 Immunofluorescence Analyzer (Basic Egg Biotechnology Co., Ltd, Nanjing, China) was used to determine the plasma BNP levels. In addition, GE Vivid E9 Ultrasound Machine (Promega, Madison, WI, USA) was used to ascertain LVEF in patients with AF. CRP >10 mg/L, TBA >10 μmol/L, BNP >900 pg/mL (age >60 years, <70 years), BNP >1800 pg/mL (age >70 years) and Hcy >15 μmol/L were defined as increase, and LVEF <50% was defined as decrease.

### 2.5 Statistical Methods

Statistical analysis was performed by SPSS 19.0 (IBM Corp., Chicago, IL, USA). The continuous variable data of normal distribution was expressed as mean ±standard deviation (SD), and Student *t*-test was applied to compare between groups. Non-normal distribution variables were represented as medians (quartiles), and Wilcox rank sum test was used for comparison between groups. Qualitative data were expressed as percentage, and comparison between groups was performed by χ^2^ test.

Shannon index and Chao abundance were calculated by R software (version 2.15.3, R Core Team, 2013, Vienna, Austria). Principal component analysis (PCA) was conducted by Facto MineR software package (version 2.15.3) in R software.

Wilcoxon rank-sum test was applied for the differential abundance of phylum, class, order, family, genus and Kyoto Encyclopedia of Genes and Genomes (KEGG) modules, furthermore, Benjamini and Hochberg methods were used for *p* values calibration and multiple tests. Lefse software (version 1.0, Huttenhower Lab, Boston, MA, USA) was employed to examine colony differences, and linear discriminant analysis (LDA) was utilized to estimate the impact of each species abundance on the difference effect. Spearman correlation analysis was employed to do correlation analysis. *p <* 0.05 was considered to be statistically significant. The data analysis processes are shown in **Supplementary Fig. 1**.

## 3. Results

### 3.1 Comparison of Microbiota Characteristics between Group AF and Group H

**Fig. 1.**
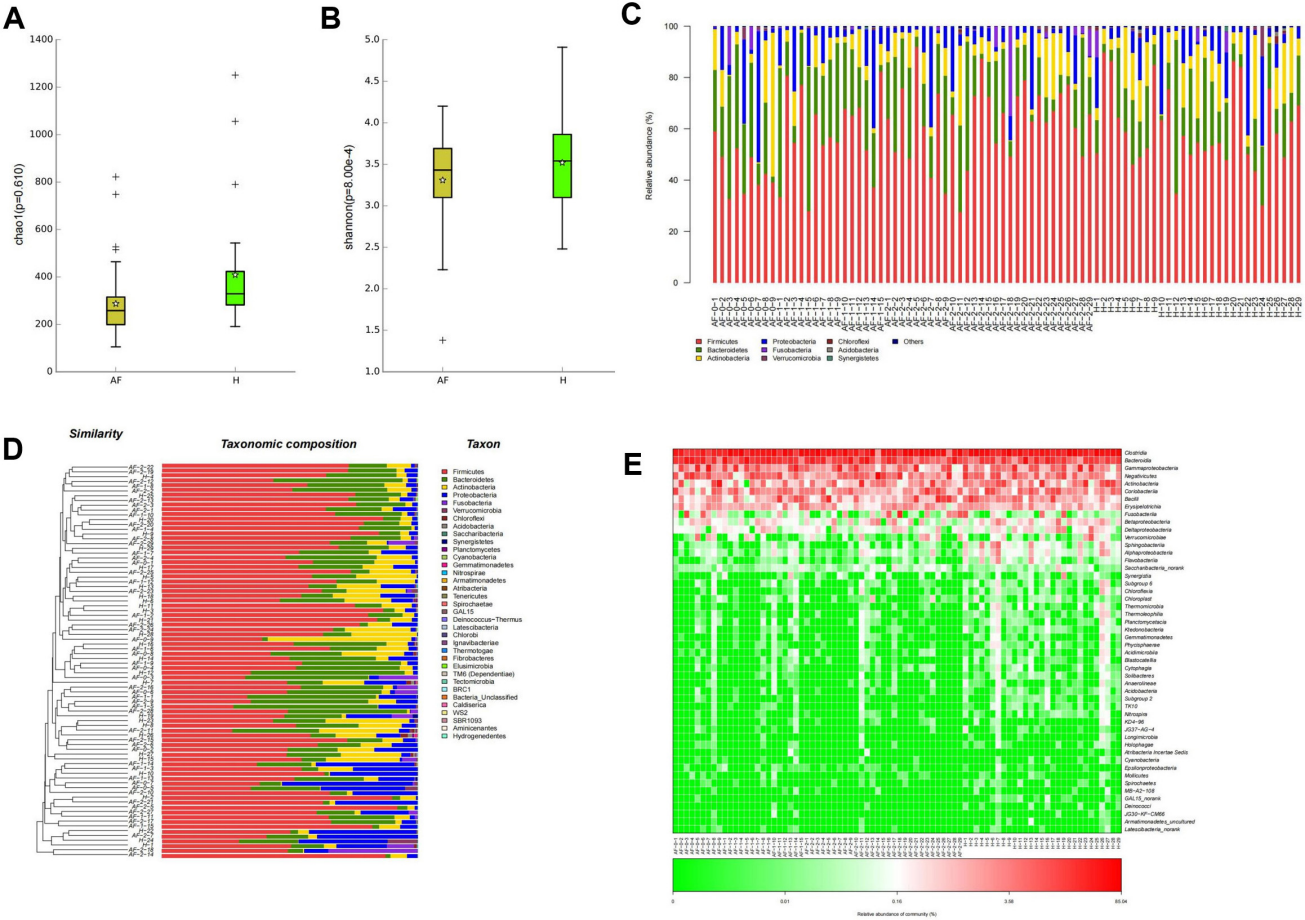
**Reduced intestinal microbial diversity in AF patients**. (A,B) The Alpha diversity of Group AF and Group H compared according to Chao1 index and Shannon index. (C) Taxonomic histogram of the top 9 species with the highest relative abundance in both Group AF and Group H at phylum level. Other indicated that microbes with abundance less than 1% were merged. The vertical axis shows the relative proportion of species and the horizontal axis shows the grouping information. (D) Combined diagram of cluster tree and community structure histogram of samples in Groups AF and H at the phylum level. The species cluster tree is on the left, and the community structure histogram is on the right. (E) Heat map of species composition at class level. AF, atrial fibrillation; H, healthy.

Intestinal microbial diversity has been regarded as a key factor in human health and diseases [14, 15]. Therefore, we analyzed the abundance (Chao1 index, Fig. 1A) and diversity (Shannon index, Fig. 1B) of the two groups via Alpha diversity index, showing that the abundance and diversity of bacteria in Group AF were largely lower than those in the control group (*p <* 0.05, Fig. 1A,B).

Then, we further analyzed the composition of intestinal flora in the two groups, showing that at phylum and class levels, there were no statistical differences in microbial community structure and composition between Group AF and Group H (*p >* 0.05, Fig. 1C–E). Hence, the outcomes ascertained that AF notably reduced the intestinal microbial abundance, but had no remarkable effect on microbial community composition.

### 3.2 Intestinal Flora Difference between Group AF and Group H and Correlation Analysis 

Subsequently, a taxonomic feature analysis was conducted to compare the taxonomic features of intestinal flora between AF patients and healthy individuals. Meanwhile, Spearman correlation analysis was used to evaluate the correlation between different classification levels of flora and age, inflammatory factors (CRP and Hcy), and cardiovascular indicators (Hs-cTn, TBA, BNP, and LVEF).

#### 3.2.1 Intestinal Flora Difference between Group AF and Group H and Correlation Analysis at Phylum Level

The results disclosed that the relative abundance of Deinococcus-Thermus in Group AF was notably lower than that in Group H (Table 1, *p <* 0.05). Furthermore, the relative abundance of Acidobacteria in Group AF was negatively correlated with CRP, with statistical difference (Table 2, *p <* 0.05).

**Table 1.** **Intestinal flora differences between Group AF and Group H at phylum level**.

Phylum	Group AF	Group H	*p*-value	q-value
Deinococcus-Thermus	6.46 × ${\mathrm{10}}^{-\,6}$ ± 3.56 × ${\mathrm{10}}^{-\,5}$	5.43 × ${\mathrm{10}}^{-\,5}$ ± 1.22 × ${\mathrm{10}}^{-4}$	6.80 × ${\mathrm{10}}^{-4}$	2.04 × ${\mathrm{10}}^{-2}$

AF, atrial fibrillation; H, healthy.

**Table 2.** **Correlation of flora in Group AF at phylum level**.

Name	env	correlation	*p*-value
Acidobacteria	CRP	–0.2845555	0.03891324

AF, atrial fibrillation; CRP, C-reactive protein; env, environment variables.

#### 3.2.2 Intestinal Flora Difference between Group AF and Group H and Correlation Analysis at Class Level

At the class level, the relative abundances of Thermoleophilia, WSP-1 (Phycisphaerae), Ktedonobacteria and Thermomicrobia in the Group AF were significantly lower than those in Group H, and the differences were statistically significant (*p <* 0.05) (Table 3). Spearman correlation analysis further demonstrated that the relative abundance of Sphingobacteriia and Thermomicrobia in the Group AF was positively correlated with LVEF, with statistical difference (*p <* 0.05) (Table 4).

**Table 3.** **Intestinal flora differences between Group AF and Group H at class level**.

Class	Group AF	Group H	*p*-value	q-value
Thermoleophilia	1.49 × ${\mathrm{10}}^{-4}$ ± 5.41 × ${\mathrm{10}}^{-4}$	6.30 × ${\mathrm{10}}^{-4}$ ± 1.57 × ${\mathrm{10}}^{-3}$	2.70 × ${\mathrm{10}}^{-4}$	2.35 × ${\mathrm{10}}^{-2}$
Phycisphaerae	7.68 × ${\mathrm{10}}^{-\,5}$ ± 2.93 × ${\mathrm{10}}^{-4}$	4.35 × ${\mathrm{10}}^{-4}$ ± 9.08 × ${\mathrm{10}}^{-4}$	2.91 × ${\mathrm{10}}^{-4}$	2.50 × ${\mathrm{10}}^{-2}$
Ktedonobacteria	1.13 × ${\mathrm{10}}^{-4}$ ± 2.93 × ${\mathrm{10}}^{-4}$	4.47 × ${\mathrm{10}}^{-4}$ ± 8.41 × ${\mathrm{10}}^{-4}$	3.32 × ${\mathrm{10}}^{-4}$	2.83 × ${\mathrm{10}}^{-2}$
Thermomicrobia	4.13 × ${\mathrm{10}}^{-\,5}$ ± 1.28 × ${\mathrm{10}}^{-4}$	8.47 × ${\mathrm{10}}^{-4}$ ± 1.46 × ${\mathrm{10}}^{-3}$	4.37 × ${\mathrm{10}}^{-4}$	3.67 × ${\mathrm{10}}^{-2}$

AF, atrial fibrillation; H, healthy.

**Table 4.** **Correlation of flora in Group AF at class level**.

Name	env	correlation	*p*-value
Sphingobacteriia	LVEF	0.3388269	0.01307315
Thermomicrobia	LVEF	0.2812475	0.04134328

AF, atrial fibrillation; LVEF, left ventricular ejection fraction; env, environment variables.

#### 3.2.3 Intestinal Flora Difference between Group AF and Group H and Correlation Analysis at Order Level

At order level, the abundances of Bacillales, Tepidisphaerales, JG30-KF-CM45 and JG30-KF-AS9 in Group AF were observably lower than those in Group H (*p <* 0.05) (Table 5). Spearman correlation analysis established that the relative abundances of JG30-KF-AS9 and Sphingobacteriales were positively correlated with LVEF, and the relative abundances of Rhodospirillales were negatively correlated with CRP, and the differences were statistically significant (*p <* 0.05) (Table 6).

**Table 5.** **Intestinal flora differences between Group AF and Group H at order level**.

Order	Group AF	Group H	*p*-value	q-value
Bacillales	5.78 × ${\mathrm{10}}^{-4}$ ± 1.35 × ${\mathrm{10}}^{-3}$	3.18 × ${\mathrm{10}}^{-3}$ ± 6.25 × ${\mathrm{10}}^{-3}$	1.07 × ${\mathrm{10}}^{-4}$	1.80 × ${\mathrm{10}}^{-2}$
Tepidisphaerales	7.43 × ${\mathrm{10}}^{-\,5}$ ± 2.85 × ${\mathrm{10}}^{-4}$	3.75 × ${\mathrm{10}}^{-4}$ ± 8.35 × ${\mathrm{10}}^{-4}$	1.50 × ${\mathrm{10}}^{-4}$	2.52 × ${\mathrm{10}}^{-2}$
JG30-KF-CM45	2.26 × ${\mathrm{10}}^{-\,5}$ ± 6.37 × ${\mathrm{10}}^{-\,5}$	8.41 × ${\mathrm{10}}^{-4}$ ± 1.44 × ${\mathrm{10}}^{-3}$	1.71 × ${\mathrm{10}}^{-4}$	2.85 × ${\mathrm{10}}^{-2}$
JG30-KF-AS9	3.23 × ${\mathrm{10}}^{-\,5}$ ± 1.30 × ${\mathrm{10}}^{-4}$	1.65 × ${\mathrm{10}}^{-4}$ ± 3.14 × ${\mathrm{10}}^{-4}$	1.93 × ${\mathrm{10}}^{-4}$	3.21 × ${\mathrm{10}}^{-2}$

AF, atrial fibrillation; H, healthy.

**Table 6.** **Correlation of flora in Group AF at order level**.

Name	env	correlation	*p*-value
JG30-KF-AS9	LVEF	0.4014989	0.002884945
Rhodospirillales	CRP	–0.2956155	0.03162799
Sphingobacteriales	LVEF	0.3388269	0.01307315

AF, atrial fibrillation; LVEF, left ventricular ejection fraction; CRP, C-reactive protein; env, environment variables.

#### 3.2.4 Intestinal Flora Difference between Group AF and Group H and Correlation Analysis at Family Level

At family level, the relative abundance of DA111 and BIrii41 bacteria in Group AF was markedly lower than that in Group H (*p <* 0.05) (Table 7). Besides, Spearman correlation analysis illustrated that abundances of Moraxellaceae and Nocardiaceae were negatively correlated with CRP, and relative abundances of Sphingobacteriaceae were positively correlated with LVEF. The differences were statistically significant (*p <* 0.05) (Table 8).

**Table 7.** **Intestinal flora differences between Group AF and Group H at family level**.

Family	Group AF	Group H	*p*-value	q-value
DA111	1.94 × ${\mathrm{10}}^{-\,6}$ ± 7.98 × ${\mathrm{10}}^{-\,6}$	1.17 × ${\mathrm{10}}^{-4}$	1.12 × ${\mathrm{10}}^{-4}$	3.91 × ${\mathrm{10}}^{-2}$
BIrii41	1.29 × ${\mathrm{10}}^{-\,6}$ ± 9.40 × ${\mathrm{10}}^{-\,6}$	7.79 × ${\mathrm{10}}^{-\,5}$ ± 2.16 × ${\mathrm{10}}^{-4}$	1.28 × ${\mathrm{10}}^{-4}$	4.45 × ${\mathrm{10}}^{-2}$

AF, atrial fibrillation; H, healthy.

**Table 8.** **Correlation of flora in Group AF at family level**.

Name	env	correlation	*p*-value
Moraxellaceae	CRP	–0.3049725	0.026386
Nocardiaceae	CRP	–0.3121728	0.02286647
Sphingobacteriaceae	LVEF	0.2893506	0.03560085

AF, atrial fibrillation; LVEF, left ventricular ejection fraction; CRP, C-reactive protein.

#### 3.2.5 Intestinal Flora Difference between Group AF and Group H and Correlation Analysis at Genus Level

However, there was no significant difference between Group AF and Group H at genus level (*p >* 0.05).

### 3.3 Microbial Community Characteristics among ${\mathrm{CHA}}_{2}$${\mathrm{DS}}_{2}$-${\mathrm{VAS}}_{\text{C}}$ Score Subgroups in Group AF

**Fig. 2.**
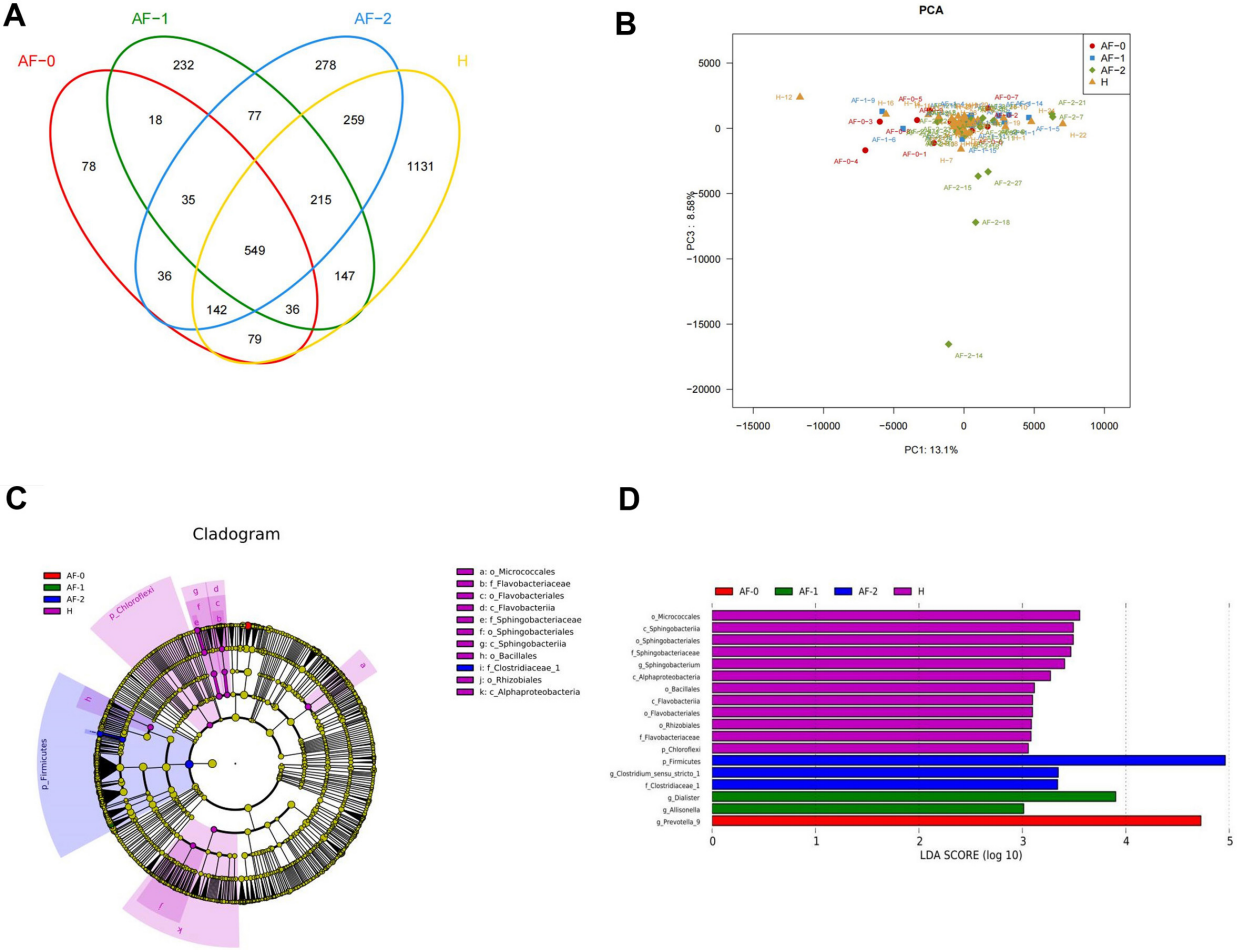
**Microbiota characteristics of ${\mathrm{CHA}}_{2}$${\mathrm{DS}}_{2}$-${\mathrm{VAS}}_{\text{𝐂}}$ scores among different subgroups in Group AF**. (A) Venn diagram of OTU between Group H and 3 subgroups (AF-0, AF-1, and AF-2) is used to analyze common genes. (B) PCA based on the abundance of OTU between Group H and 3 subgroups (AF-0, AF-1 and AF-2). Horizontal axis: first principal component (PC1: 13.1%), vertical axis: second principal component (PC3: 8.58%). (C) Lefse differential analysis was used to analyze the differences in bacterial classification between Group H and the 3 subgroups (AF-0, AF-1 and AF-2). (D) Based on the classification information, LDA analysis was performed on the microbial groups that had significant interaction between Group H and 3 subgroups (AF-0, AF-1 and AF-2). LDA value ($\log_{10}$) >4. AF, atrial fibrillation; H, healthy; LDA, linear discriminant analysis

Previous studies have shown that ${\mathrm{CHA}}_{2}$${\mathrm{DS}}_{2}$-${\mathrm{VAS}}_{\text{C}}$ score can be used to predict cardiac embolism in patients with AF [4]. Therefore, in terms of ${\mathrm{CHA}}_{2}$${\mathrm{DS}}_{2}$-${\mathrm{VAS}}_{\text{C}}$ score, Group AF was divided into three subgroups: 0 point (AF-0), 1 point (AF-1) and ≥2 points (AF-2), and the characteristics of fecal flora among different subgroups were analyzed. First, we draw Venn diagram by OTU cluster analysis (Fig. 2A), whose outcomes exhibited that three subgroups and the Group H shared 79 OTUs. Among them, 78, 232, 278 and 1131 specific OTUs were found in Group AF-0, AF-1, AF-2 and H, respectively. Next, we performed PCA analysis on three subgroups and Group H, which reported that Group H, AF-0 and AF-1 were relatively concentrated in the middle, while Group AF-2 was relatively dispersed (Fig. 2B). Subsequently, we applied Lefse software to analyze the colony differences between Group H and three subgroups AF-0, AF-1 and AF-2. At the same time, linear discriminant analysis (LDA) was utilized to estimate the impact of each species abundance on differential effects. Fig. 2C showed that Firmicutes in Group AF-2 and Chloroflexi in Group H were relatively more abundant at phylum level. At class level, the relative abundance of Sphingobacteriia, Flavobacteriia and Alphaproteobacteria was higher in Group H. At order level, the relative abundance of Sphingobacteriales, Micrococcales, Flavobacteriales, Sphingobacteriales and Rhizobiales in Group H was higher. At family level, the relative abundance of Sphingobacteriaceae, Flavobacteriaceae and Clostridiaceae was higher in Group H. In Fig. 2D, it can be seen at genus level, the relative abundance of *Sphingobacterium* in Group H, *Clostridiumsensustricto-1* in Group AF-2, Dialister and *Allisonella* in Group AF-1 and *Prevotella-9* in Group AF-0 were higher. The LDA scores of Firmicutes at phylum level and *Prevotella-9* at genus level were higher, both >4.

## 4. Discussion

### 4.1 AF and Intestinal Flora

There is no denying that cardiovascular diseases (CVDs) are the leading cause of death worldwide [16]. In recent years, the role of the gut microbiome in CVDs has received much attention [17]. The homeostasis of gut microbiota plays an essential role in maintaining the growth of pathogenic microbes in healthy people [18]. Conversely, dysfunction of gut microbiota often leads to inflammatory bowel disease (IBD), obesity, diabetes, colorectal cancer, and CVD such as hypertension, heart failure, and atherosclerosis [18, 19, 20]. Among them, previous studies have established that obesity, hypertension, diabetes and atherosclerosis are risk factors for AF [21, 22]. In addition, dysregulation of gut microbiota-derived metabolites such as TMAO may also induce CVD [20]. Therefore, the purpose of this study is to explore the changes of flora in patients with AF, analyze its correlation with ${\mathrm{CHA}}_{2}$${\mathrm{DS}}_{2}$-${\mathrm{VAS}}_{\text{C}}$ score, and explore the related factors that may affect the changes of intestinal flora.

### 4.2 CRP/LVEF Values and Intestinal Flora 

CRP is a very important non-specific inflammatory transmitter in the human body, which has been proved to be the most predictable indicator of vascular inflammation and is related to AF, but the root cause is not clear. At present, numerous studies have revealed that the occurrence and recurrence of AF are closely related to inflammatory factors, and inflammation may be related to myocardial remodeling in AF [23]. Chung* et al*. [24] first reported that elevated CRP level is associated with AF using a case-control study in 2001. Takashi Koyama *et al*. [25] enrolled 186 patients with paroxysmal AF who underwent AF ablation due to poor drug treatment. The body temperature and CRP were notably higher in patients with recurrence of AF (within 3 days after surgery) than in baseline levels. Pericarditis occurred in 15 (33%) of the 45 patients with recurrence. In this study, we found that patients with AF had intestinal flora imbalance, meanwhile, the relative abundance of Acidobacteria, Rhodospirillales, Moraxellaceae and Nocardiaceae was negatively correlated with CRP. Recent studies have shown that TMAO, a metabolite derived from gut microbes, is associated with the occurrence, development and recurrence of AF [26, 27]. TMAO can regulate the level of pro-inflammatory factors by activating a variety of pro-inflammatory signaling pathways, and can induce the occurrence of AF by aggravating myocardial fibrosis [9, 26, 28]. Therefore, we speculated that the changes in CRP levels may be regulated by TMAO, which still needed a number of studies for further confirmation.

LVEF is an important index for the evaluation of heart failure. The lower the value of LVEF, the worse the systolic function and the more severe the condition of heart failure. Heart failure can lead to intestinal congestion, peripheral vascular contraction, cause intestinal microcirculation disturbance, impaired intestinal epithelial cells and permeability changes, which not only makes toxic substances easier to enter the body cycle, aggravates systemic inflammatory response, but also reduces the intestinal absorption capacity of sugar and protein, leading to malnutrition, and in turn aggravates heart failure [29, 30]. Recent studies have shown that the intestinal microbiota in patients with heart failure is dysregulated, with a distinct decrease in Faecalibacterium prausnitzii, and an obvious increase in Gastrococcus, Salmonella, Shigella, and Campylobacter jejuni [12, 31]. This study found that the relative abundances of Sphingobacteriia, Thermomicrobia, JG30-KF-AS9, Sphingobacteriaes and Sphingobacteriaceae were positively correlated with LVEF. Hence, the study further demonstrated the correlation between CRP level and LVEF value in heart failure and intestinal flora, and provided a new idea for us to further study the relationship between AF and inflammatory factors.

### 4.3 ${\mathrm{CHA}}_{2}$${\mathrm{DS}}_{2}$-${\mathrm{VAS}}_{\text{C}}$ Score and Intestinal Flora

${\mathrm{CHA}}_{2}$${\mathrm{DS}}_{2}$-${\mathrm{VAS}}_{\text{C}}$ score is widely used to assess the risk of cardiogenic thrombosis in AF patients, and the indicators include heart failure, hypertension, age, diabetes, stroke, vascular disease and gender [32]. Studies have suggested that the above indicators are related to changes in intestinal flora [33, 34, 35].

Intestinal flora is associated with several scoring points of ${\mathrm{CHA}}_{2}$${\mathrm{DS}}_{2}$-${\mathrm{VAS}}_{\text{C}}$. Chen *et al*. [36] studied the specific types of symbiotic flora associated with coronary heart disease (CHD) by systematically reviewing prospective observational studies to evaluate the relationship between symbiotic flora and CHD. Of the 544 published articles identified in the preliminary search, 16 articles from 16 cohort studies (2210 patients) were included in the analysis. Comprehensive data showed that in the fecal samples of patients with CHD, Bacteroides and Prevotella are generally identified in 9 articles, and Firmicutes are generally identified in 7 articles. Besides, in 16 cohort studies, several symbiotic bacteria are common in atherosclerotic plaques and blood or intestinal samples. For example, Veillonella, Proteobacteria and Streptococcus can be identified in plaque and feces samples, while Clostridium is common in blood and feces samples of patients with CHD, which indicates that several symbiotic bacteria are related to CHD, and their existence may be related to the disease markers of CHD.

Type-1 diabetes mellitus is a chronic metabolic disease characterized by insulin resistance, accompanied by low-level inflammation, which is closely related to substance and energy metabolism. However, there is a relationship between intestinal flora and host in regulating energy balance and inflammatory response [37]. Since the study on intestinal flora of diabetic patients was first reported in 2020, more and more research pieces of evidence have shown that there are changes in intestinal flora in diabetic patients [38]. The abundances of Clostridium and Firmicutes in diabetic patients are significantly decreased, in which the decreased abundance of butyrate-producing bacteria is particularly related to diabetes, and the decrease in butyrate is proved to be positively related to diabetes [39]. Intestinal flora participates in the occurrence of diabetes and insulin resistance by regulating inflammation, immunity and metabolism [40].

Benakis *et al*. [41] induced intestinal flora imbalance in mice by using antibiotics, and found that it can reduce acute brain injury. The mechanism may be the increase of regulatory T cells (Treg) and the decrease of IL-17 T cells. Treg plays a protective role in the brain by down-regulating the inflammatory response of ischemic brain tissue. After stroke, the intestinal flora can shift to the surrounding tissues and organs outside the gastrointestinal tract, resulting in bacterial infection, affecting the degree of damage and prognosis of stroke [42]. Patients with ischemic stroke often have complications such as microbial imbalance and constipation, while intestinal microbial imbalance affecting the progression of ischemic stroke and patient prognosis.

Our previous study [12] reported that flora of elderly patients with non-valvular AF had its own characteristics compared with Group H, and the difference may be related to the scores of ${\mathrm{CHA}}_{2}$${\mathrm{DS}}_{2}$-${\mathrm{VAS}}_{\text{C}}$, hypertension and permanent AF. As what have been revealed, there was a consistent trend between the number of bacteria and ${\mathrm{CHA}}_{2}$${\mathrm{DS}}_{2}$-${\mathrm{VAS}}_{\text{C}}$ score, and meaningful differences in the number of Faecalibacterium prausnitzii, Bacteroides and Clostridium leptum between the low, middle and high subgroups of the score, indicating that ${\mathrm{CHA}}_{2}$${\mathrm{DS}}_{2}$-${\mathrm{VAS}}_{\text{C}}$ score was correlated with the change of flora. ${\mathrm{CHA}}_{2}$${\mathrm{DS}}_{2}$-${\mathrm{VAS}}_{\text{C}}$ score was negatively correlated with Bacteroides and Clostridium leptum (*p <* 0.05), which may be used as an indicator of the changes of flora in patients with AF and explore a new direction for the treatment of AF.

In this study, bioinformatics analysis of ${\mathrm{CHA}}_{2}$${\mathrm{DS}}_{2}$-${\mathrm{VAS}}_{\text{C}}$ score in Group AF and Group H displayed that the relative abundance of Firmicutes in Group AF-2 and Chloroflexi in Group H was higher at the phylum level. At class level, the relative abundance of Sphingobacteriia, Flavobacteriia and Alphaproteobacteria was higher in Group H. At order level, the relative abundances of Sphingobacteriales, Micrococcales, Flavobacteriales, Sphingobacteriales and Rhizobiales in Group H were higher. At family level, the relative abundance of Sphingobacteriaceae, Flavobacteriaceae and Clostridiaceae was higher in Group H. At genus level, the relative abundances of *Sphingobacterium* in Group H, *Clostridiumsensustricto-*1 in the group with ${\mathrm{CHA}}_{2}$${\mathrm{DS}}_{2}$-${\mathrm{VAS}}_{\text{C}}$ score ≥2, *Dialister* in the group with ${\mathrm{CHA}}_{2}$${\mathrm{DS}}_{2}$-${\mathrm{VAS}}_{\text{C}}$ score 1, *Allisonella* in the group with ${\mathrm{CHA}}_{2}$${\mathrm{DS}}_{2}$-${\mathrm{VAS}}_{\text{C}}$ score ≥2, *Prevotella-9* in the group with ${\mathrm{CHA}}_{2}$${\mathrm{DS}}_{2}$-${\mathrm{VAS}}_{\text{C}}$ score 0 was higher.

As can be seen from the histogram of Lefse differential analysis that the LDA scores of Firmicutes in the group with ${\mathrm{CHA}}_{2}$${\mathrm{DS}}_{2}$-${\mathrm{VAS}}_{\text{C}}$ score ≥2 at phylum level and *Prevotella-9* in the group with ${\mathrm{CHA}}_{2}$${\mathrm{DS}}_{2}$-${\mathrm{VAS}}_{\text{C}}$ score 0 at genus level were the highest, all >4. Recent studies have reported that the bacteria in human intestinal tract mainly belong to the following five phyla: Firmicutes, Bacteroidetes, Actinomycetes, Proteobacteria, and Verrucomicrobia, among which, Firmicutes and Bacteroidetes account for more than 90% of the total intestinal microorganisms, while the other phyla account for less than 1% of the total intestinal microorganisms [43]. Many studies have shown that Firmicutes are closely related to cardiovascular disease. Cui *et al*. [44] analyzed the intestinal flora of healthy volunteers and patients with CHD, and found that the proportion of Firmicutes in CHD group was higher. In another study, a rat model of hypertension treated with long-term angiotensin infusion showed a significant decrease in the richness of the microbiota and a significant increase in the ratio of Firmicutes to bacteroides [45].

*Prevotella-9 *is a common non-spore Gram-negative anaerobic bacterium, which is a new genus isolated from Bacteroides in recent years, including 20 species, the most common of which is *P.melaninogenica1*. It is a common opportunistic pathogen in clinic, which can cause endogenous infection in female genital tract and oral cavity. The relative abundance of *Prevotella-9* in group with ${\mathrm{CHA}}_{2}$${\mathrm{DS}}_{2}$-${\mathrm{VAS}}_{\text{C}}$ score 0 is high, but there are few reports on *Prevotella-9* and cardiovascular diseases, whose clinical significance needs to be further studied.

## 5. Conclusions

${\mathrm{CHA}}_{2}$${\mathrm{DS}}_{2}$-${\mathrm{VAS}}_{\text{C}}$ score is correlated with the change of flora, which may be used as an indicator of microbiota changes in patients with AF, providing a new direction for the treatment of AF patients with different ${\mathrm{CHA}}_{2}$${\mathrm{DS}}_{2}$-${\mathrm{VAS}}_{\text{C}}$ scores by improving intestinal flora. However, due to the sample size of this study and the complex interaction between patients’ diseases, the relevant conclusions still need to be further confirmed by increasing the sample size.

## Data Availability

The datasets used and/or analyzed during the current study are available from the corresponding author on reasonable request.

## Supplementary Material

Supplementary material associated with this article can be found, in the online version, at https://doi.org/10.31083/j.rcm2404110.
