# Supplementary figures and images for: Changes of Intestinal Flora in Patients with Atrial Fibrillation and Its Correlation with Cardiovascular Risk Factors
Source: Rev Cardiovasc Med. 2023 Apr 17;24(4):110. doi: 10.31083/j.rcm2404110 (PMC11273065; doi:10.31083/j.rcm2404110)

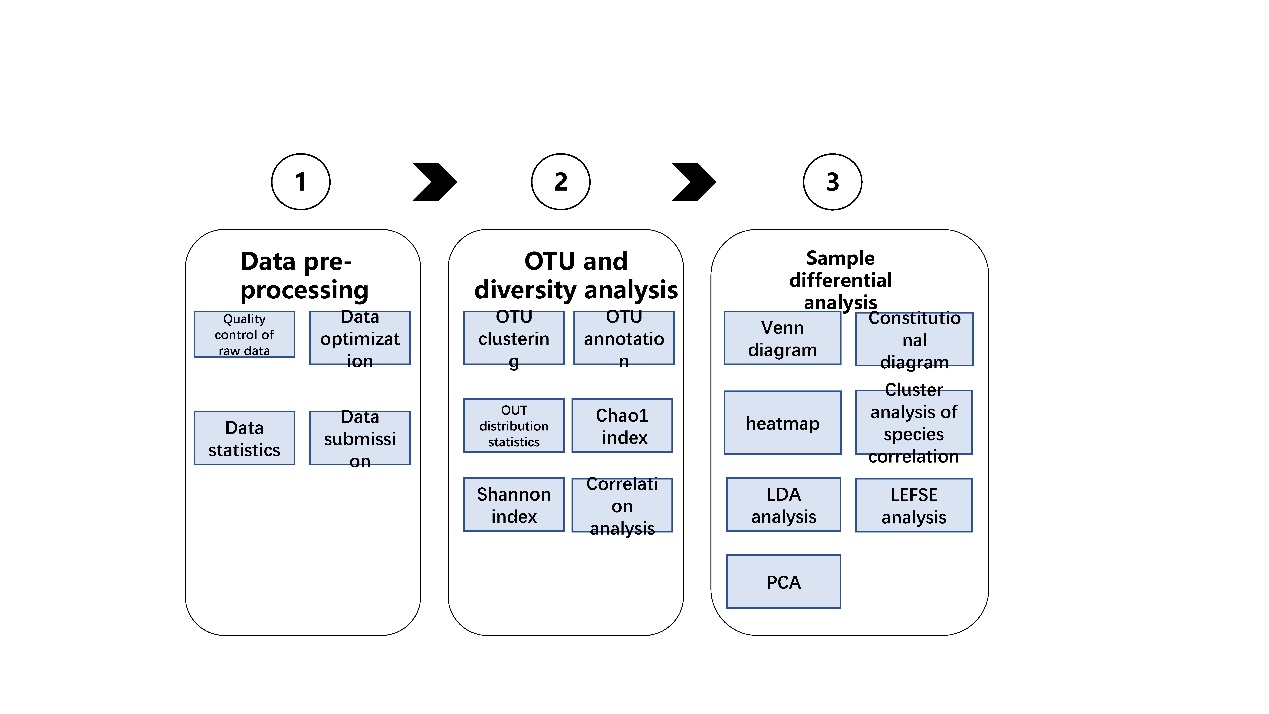


Supplementary Fig. 1. Flow chart of data analysis methods.

Supplement: Supplementary file 1 [file 2153-8174-24-4-110-s1.zip › 2153-8174-24-4-110-s1.docx]
